# Fabrication of a Cation-Exchange Membrane via the Blending of SPES/N-Phthaloyl Chitosan/MIL-101(Fe) Using Response Surface Methodology for Desalination

**DOI:** 10.3390/membranes12020144

**Published:** 2022-01-25

**Authors:** Xiaomeng Wang, Qun Wang, Mengjuan Zhao, Lu Zhang, Xiaosheng Ji, Hui Sun, Yongchao Sun, Zhun Ma, Jianliang Xue, Xueli Gao

**Affiliations:** 1 College of Chemical and Biological Engineering, Shandong University of Science and Technology, Qingdao 266590, China; 15764237673@163.com (X.W.); 18561555725@163.com (Q.W.); zmj971830@163.com (M.Z.); luluzh5709@163.com (L.Z.); 2 Sanya Institute of Oceanology, Chinese Academy of Sciences, Sanya 572000, China; 3 State Key Laboratory of High-Efficiency Utilization of Coal and Green Chemical Engineering, Ningxia University, Yinchuan 750021, China; sunhui@nxu.edu.cn; 4 Key Laboratory of Marine Chemistry Theory and Technology, Ministry of Education, College of Chemistry and Chemical Engineering, Ocean University of China, Qingdao 266100, China; yongchao_sun@163.com (Y.S.); gxl_ouc@126.com (X.G.); 5 School of Chemical Engineering, Dalian University of Technology, Dalian 116024, China; 6 College of Safety and Environmental Engineering, Shandong University of Science and Technology, Qingdao 266590, China; ll-1382@163.com

**Keywords:** cation exchange membrane, response surface methodology, membrane synthesis, electrodialysis

## Abstract

In the present work, a novel mixed matrix cation exchange membrane composed of sulfonated polyether sulfone (SPES), N-phthaloyl chitosan (NPHCs) and MIL-101(Fe) was synthesized using response surface methodology (RSM). The electrochemical and physical properties of the membrane, such as ion exchange capacity, water content, morphology, contact angle, fixed ion concentration and thermal stability were investigated. The RSM based on the Box–Behnken design (BBD) model was employed to simulate and evaluate the influence of preparation conditions on the properties of CEMs. The regression model was validated via the analysis of variance (ANOVA) which exhibited a high reliability and accuracy of the results. Moreover, the experimental data have a good fit and high reproducibility with the predicted results according to the regression analysis. The embedding of MIL-101(Fe) nanoparticles contributed to the improvement of ion selective separation by forming hydrogen bonds with the polymer network in the membrane. The optimum synthesis parameters such as degree of sulfonation (DS), the content of SPES and NPHCs and the content of MIL-101(Fe) were acquired to be 30%, 85:15 and 2%, respectively, and the corresponding desalination rate of the CEMs improved to 136% while the energy consumption reduced to 90%. These results revealed that the RSM was a promising strategy for optimizing the preparation factors of CEMs and other similar multi-response optimization studies.

## 1. Introduction

RSM integrating mathematics with statistics has been widely utilized to investigate, optimize and model the performance of complex systems, which can determine the significant factors affecting an experiment initiating from the design of experiment (DOE). It aims at reducing the number of experimental runs while maximizing output through the data generated. The data can be used to develop a regression model, which facilitates the acquirement of optimal parameters. There are three experimental design methods including the central composite design (CCD), Box–Behnken design (BBD) and the Doehlert Matrix (DM). BBD composed of three interlocking 2^2^ factorial designs with some points is one of the most popular spherical and revolving RSM designs, which is applied widespread for optimizing parameters. The RSM has been widely used in designing experiments, developing regression models and determining optimal variables in chemical, physical, biological and environmental processes [1]; however, as far as we are concerned, few studies have been conducted on the optimizing and modeling of the interaction effects of the synthetic parameters of a mixed matrix cation exchange membrane (MMCEMs).

The scarcity of potable water has been a crucial issue in the world due to population growth and economic globalization, which cause over-exploitation and contamination of freshwater resources by industry, agriculture and urbanization [2,3]. Of note is that more than 96.5% of the total global water resources are salty, whereas only 0.6% of it can be utilized as potable water. Consequently, the desalination process is a quite feasible approach to produce fresh water to resolve the serious problem of water resource shortages. As a membrane-based water purification technology, ion exchange membranes (IEMs), especially the cation exchange membranes (CEMs), are widely applied to seawater desalination, wastewater reuse [4,5,6], fuel cells [7], food processing and other fields [8,9,10,11,12] due to their high recovery, efficiency, environmental friendliness, low energy cost and easy operation; however, a lower ion conductivity, high resistance and poor permselectivity hinder the application of the CEMs. In order to resolve this crucial problem, variation of the polymeric matrix, functional groups and additives are employed to improve the desirable properties of the CEMs for different applications [13,14,15].

Recently, the primary materials used in synthetic CEMs have been polymers such as polysulfone (PS), polyethersulfones (PES), sulfonated polyethersulfones (SPES), polyvinyl alcohol (PVA), poly(2,6-dimethyl-1,4-phenyleneoxide) (PPO), sulfonated poly(ether ether ketone) (SPEEK) and so on [16,17]. Among these polymers, SPES with an ionizable functional group has been developed as a desirable polymeric backbone for CEMs because of its low fabrication costs and high mechanical flexibility. These fixed charge groups enhance the hydrophilicity and electrostatic interactions with ions, which is a significant factor determining the ability of CEMs to selectively reject some ions (e.g., anions) but not others (e.g., cations). Chitosan (CS) extracted from the exoskeletons of crustaceans contains amino groups (-NH_2_) and hydroxyl groups (-OH) that bestow the high hydrophilicity of CS. Moreover, CS attracts much more attention for different applications such as food packaging, drug delivery and IEMs, due to the advantages of its cost-effectiveness, biological compatibility and environmental benefits. In our previous work [18], SPES/N-phthaloyl chitosan (NPHCS) had been developed for improving the physicochemical properties of CEMs; however, the ion exchange capacity (IEC) and ionic conductivity of the prepared CEMs improved only slightly. At present, more versatile strategies have been investigated for incorporating non-ion exchange additives into the polymetric backbone in order to enhance the properties of CEMs.

Metal organic frameworks (MOFs), consisting of metal ions coordinated to organic ligands as linkers have been studied as a new category of organic/inorganic hybrid materials with a tailored pore structure [19,20]. Additionally, the organic linkers present in the MOFs’ structures were studied to provide a better affinity with the MOF and organic polymers, compared with purely inorganic compounds as an additive that has been utilized for the preparation of advanced mixed matrix membranes [21,22]. The good compatibility between these two phases have been related to the formation of non-covalent bonds, such as hydrogen bonds, or even covalent bonds when the MOF–polymer composite is formed via in situ polymerization. This nature provides an opportunity for improving the mechanical and thermal stabilities of the hybrid membranes, as well as their ionic conductivity [23]. To sum up, incorporating MOFs in hybrid ion exchange membranes would acquire unique advantages over other nanostructure materials [24,25]. Incorporating acid-grafted inorganic fillers into the polymer matrix can increase the proton conductivity and interfacial compatibility of the composite membranes. Based on the abovementioned characteristics, the MOF-based mixed matrix ion exchange membranes have attracted much more attention because of their improvement of IEC, ionic conductivity and interfacial compatibility [6,18,26,27]. The current state of knowledge reveals that the combination of MIL-101(Fe) composite nanoparticles and an acidic functionalized polymer matrix can utilize each of their strengths to improve the performance abilities [28,29,30]. For constructing a MIL-101(Fe)-based composite membrane with flexible and mechanical ability, classic and easy-to-operate methods were adopted to ensure the dispersion of MIL-101(Fe) nanoparticles within the whole of the acidic functionalized polymer matrix. Because of hydrophilic–hydrophilic interactions, MIL-101(Fe) was expected to be confined within the hydrophilic pore/ion conducting ways of the membrane matrix, which is responsible for the pore modification.

Herein, a novel hybrid CEM with multi-dimensional nanocomposite structures by embedding MIL-101(Fe) into the SPES/NPHCs polymetric backbone was synthesized by response surface methodology. The interaction effect of three independent and crucial factors (DS, the ratio of SPES, NPHCs, and MIL-101(Fe) content) on the properties of CEMs were investigated by a Box–Behnken design model. The predicted results were exhibited from three-dimensional response surface plots to validate the interactive effect of synthesized parameters on the properties of the CEMs. The veracity of the regression model between the predicted results and experimental data was confirmed by ANOVA.

## 2. Materials and Methods

### 2.1. Materials

N,N-dimethylformamide (DMF), acetic acid and methanol were purchased from Chengdu Cologne Chemical Co., Ltd. (Chengdu, China). Terephthalic acid, FeCl_3_∙6H_2_O, phthalic anhydride, dichloromethane and N-methylpyrrolidone (NMP) were supplied by Macklin Biochemical Co., Ltd. (Shanghai, China). Chitosan (80–95% degree of deacetylation), chlorosulfonic acid and polyethersulfone (PES, molecular weight was about 50,000) were provided by Sinopharm Chemical Reagent Co., Ltd. (Shanghai, China). All reagents and solvents were analytical grade. All the solutions were prepared using pure water.

### 2.2. Synthesis of MIL-101(Fe)

The MIL-101(Fe) was synthesized according to a hydrothermal method which followed similarly the description of Balu et al. with a corresponding adjustment [31]. The terephthalic acid, FeCl_3_∙6H_2_O and acetic acid were gradually added to 90 mL of N, N-dimethylformamide and treated with an ultrasonic treatment for 30 min to obtain an evenly dispersed mixture. The mixture was transferred into a Teflon-lined stainless-steel bomb and heated at 110 °C for 12–24 h. The brown powder was obtained by centrifugation after the hydrothermal reaction process. The powder was thoroughly washed three times with DMF and methyl alcohol. Afterwards, the mixture was centrifuged and dried at 80 °C for 24 h under vacuum. In order to adjust the suitable size of the MIL-101(Fe) for the membranes and to optimize the operational parameters, the reaction parameters were optimized by BBD methodology and determined to be 1.07 g FeCl_3_·6H_2_O, 0.49 g terephthalic acid, 3.18 mL acetic acid and 18 h.

### 2.3. Preparation of Sulfonated Polyethersulfone and N-Phthaloyl Chitosan

The drafting of the flexible sulfonic acid group onto the polymer was implemented according to a procedure that has been reported in previous work. In a 200 mL three-necked reaction flask with a nitrogen atmosphere, polyether sulfone (PES, 4 g) was stirred until the solute has dissolved in dichloromethane (50 g) at 25 °C. While the homogeneous solution formed and kept stirring, the chlorosulfonic acid (5–10 mL) was added into the homogeneous solution drip by drip at a constant speed from a constant pressure of a funnel. After full stirring and reaction, a mixture was formed, then the mixture was quickly immersed in ice water with agitation. The solid products were collected and washed by DI water until the pH was approximately 6–7. Then, the obtained sulfonated polyether sulfone was dried at 60 °C for 48 h in a vacuum oven, thus, the synthetic path of the sulfonation polyethersulfone is presented in Figure S1.

The synthesis of the N-phthaloyl chitosan (NPHCs) was performed according to a procedure reported previously. In a 200 mL reaction flask with a nitrogen atmosphere, the chitosan (1 g) and phthalic anhydride (4.48 g) were stirred until the solute was dissolved in the DMF (20 mL). The solution was heated to 130 °C for 6 h. Then, the viscous product was quickly immersed in ice water with agitation. The solid products were collected and purified by ethanol and ethyl ether. Finally, the product was dried at 40 °C for 24 h under vacuum. The synthetic path of the N-phthaloyl chitosan is shown in Figure S2.

### 2.4. Synthesis of Ion Exchange Membranes

**Figure 1:**
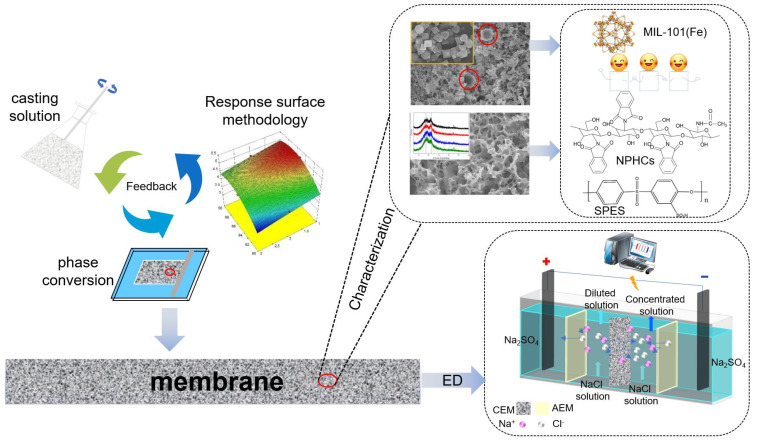
Schematic of the synthesis of the anion-exchange membrane via blending of SPES/N-phthaloyl chitosan/MIL-101(Fe).

The MIL-101(Fe)-sulphonated poly (ether sulfone) membrane was prepared using sulfonated polyether sulfone (SPES), N-phthaloyl chitosan (NPHCs) and MIL-101(Fe) [32,33,34,35]. Firstly, the SPES and NPHCs were added in the NMP with a nitrogen atmosphere. The solution was heated to 60 °C for 6 h and stirred until the solute dissolved. Then, the MIL-101(Fe) nanoparticles were added into the solution and stirring continued for 6 h. In order to ensure the nanoparticles were fully dispersed in the viscous solution, the solution was ultrasonicated for 45 min, then set for 24 h. The MIL-101(Fe)-sulphonated poly (ether sulfone) membrane was formed by casting the viscous solution on a glass plate and spreading it in a homogeneous liquid state. The product was dried at 100 °C for 24 h with air and then dried at 40 °C for 10 h under vacuum. Finally, the MIL-101(Fe)-sulphonated poly (ether sulfone) membrane was immersed in DI water to facilitate separation from the glass plant surface. The synthetic process of the SPES/NPHCs/MIL-101(Fe) CEMs has been presented in Figure 1.

### 2.5. Membrane Characterization

#### 2.5.1. Characterization Method

Powder X-ray diffraction (XRD) patterns of the MIL-101(Fe) and SPES/NPHCs membrane were obtained using a Rigaku Ultima IV X-ray Diffractometer (Tokyo, Japan) operated at 3 kW. The diffractograms were recorded in the scanning angle (2θ) range 5–40° with a step size of 0.02° and scanning velocity of 6 °/min. SEM (Hitachi S4800, Tokyo, Japan) was used to investigate the cross-sections and surface morphologies of the CEMs and MIL-101(Fe) at an accelerating voltage of 10 kV and a magnification of 1.00 k [36,37]. An ATR-FTIR Spectrometer (Nicolet 380, Madison, WI, USA) was performed to analyze the functional groups of the CEMs and other materials. The spectra were recorded from 4000 to 400 cm^−1^ by averaging 32 scans with a resolution of 4 cm^−1^. The thermal stability of the membranes was measured by a differential scanning calorimeter (Mettler Toledo DSC 1, Greifensee, Switzerland) from 30 °C to 300 °C under a nitrogen atmosphere at a heating rate of 8 °C∙min^−1^ [38,39,40]. The mechanical strength of the membrane (25 cm^2^ rectangular pieces) was measured by using a controlled tensile testing machine model AI-7000-LA supported by Geotech Testing Machines Co., Ltd. (Taichung, China).

#### 2.5.2. Water Content and Hydrophilicity

The membranes were immersed in DI water for 24 h. Then, the wet membranes were quickly wiped by filter paper and weighted (*m*_1_, g). Subsequently, the wet membranes were dried overnight and re-weighted (*m*_2_, g) as the dry weight of the membrane. To reduce the experimental error, each sample was measured three times. Water content was obtained from Equation (1):

(1) $$\mathrm{water}\;\mathrm{content}\left(\%\right)=\frac{m_{1}-m_{2}}{m_{2}}\times 100\%$$

The hydrophilicity of the CEMs was evaluated by a contact angle using the fixed droplet method and a contact angle measuring instrument (Kruss DSA30, Hamburg, Germany). The droplet (DI water, 3 μL) dripping on the membrane surface in the dosing model and the contact angle was captured after 5 s. The measurement was repeated at least 5 times for each sample and then the average value was used. All experiments were performed at room temperature.

#### 2.5.3. Ion Exchange Capacity and Fixed Ion Concentration

Ion exchange capacity (IEC) for the membranes were determined by the back-titration method. The patch of dried membrane was immersed in a HCl solution (1 mol/L) for 48 h and then removed. The remaining HCl solution was titrated by the calibrated NaOH solution until the acid and alkali neutralized. The IEC was determined by the following formula:

(2) $$\mathrm{IEC}=\frac{C_{\mathrm{HCl}}\times V_{\mathrm{HCL}}-C_{\mathrm{NaOH}}\times V_{\mathrm{NaOH}}}{m_{2}}$$

where C_HCL_, V_HCl_, C_NaOH_ and V_NaOH_ are the concentrations and volumes of the HCl and NaOH solution, and *m*_2_ is the weight of the dried membrane sample.

The fixed ion concentration (FIC) was calculated by the following formula:

(3) $$\mathrm{FIC}=\frac{\mathrm{IEC}}{\mathrm{water}\;\mathrm{content}}$$

#### 2.5.4. Diffusion Coefficient and Electrochemical Properties of the Membrane

The diffusion coefficient reflects the ability of the electrolyte to pass through the membrane under the effect of concentration, which has a certain correlation to the membrane porosity. The scheme displays the setup used for the measurement of the diffusion coefficient. The ion transport from the chamber II (NaCl solution) to chamber I, which was measured with a conductivity meter ((DDS-307, Shanghai INESA Scientific Instrument Co., Ltd., Shanghai, China), was connected to a computer. The membrane desalination performance was measured in two compartment cells, with an effective area separating the two with vertical membrane of 3.0 cm^2^ NaCl solution. The process is shown in Figures S3 and S4. The diffusion coefficient was calculated by the following formula:

(4) $$K_{s}=\frac{D}{t_{m}}=\frac{V\theta K}{S\Delta C_{0}}$$

where *K_s_* is the permeability coefficient of sodium chloride; *D* is the diffusion coefficient of sodium chloride; *t_m_* is the thickness of the membrane; *v* is the volume of sodium chloride solution; *θ* shows the linear relation between conductivity and content in chamber I; *K* shows the linear relation between time and content in chamber II; s is the effective area of membrane; and ∆*C*_0_ is the concentration difference between chamber I and chamber II.

## 3. Results and Discussion

### 3.1. Characterization of MIL-101(Fe)

It was necessary to test the stability of the MOFs in a water, weak acid and alkaline environment in order to verify the feasibility of the synthesized MOFs used in the water treatment process. At room temperature, the MIL-101(Fe) materials were immersed in dilute hydrochloric acid (pH = 5) and dilute sodium hydroxide solution (pH = 9), respectively. In deionized water, the MIL-101(Fe) was kept immersed for two days, then rinsed with a large amount of deionized water for 15 min after the immersion. This was repeated three times, then it was dried in a vacuum oven at 40 °C for 24 h, followed by the detection of the MOFs after immersion in the different environments by XRD. As shown in Figure S5, the structure of the MOFs’ skeletons exhibited good stability.

The characteristic peaks that appeared at 8.0°, 8.5°, 18.5°, 18.0°, and 21.3° are in agreement with the literature [41]. Figure S6 displays the SEM images of the MIL-101(Fe) in optimum conditions. It could be observed that the surface morphology of the MIL-101(Fe) synthesis comprised smooth particles with spindle and irregular polyhedral shapes.

Figure S7 exhibits the FTIR spectra of the MIL-101(Fe). The peak of the F-O stretching mode was observed at 533 cm^−1^, implying the existence of a metal-oxo bond between the carboxylic group of terephthalic acid and Fe^3+^. The peak of the C-H bending vibrations of benzene in the organic linkers was surveyed at 745 cm^−1^. The two intense peaks at 1388 cm^−1^ and 1535 cm^−1^ were attributed to the asymmetric and symmetric stretching of the carboxyl groups and illustrated the existence of the dicarboxylic acid anion cross-linking agent in the MIL-101(Fe). Moreover, the peak at 3432 cm^−1^ belonged to the O-H stretching vibrations of the water molecules adsorbed on the surface.

### 3.2. Characterization of Polymer Structure-Morphology (SPES and NPHCs)

**Figure 2:**
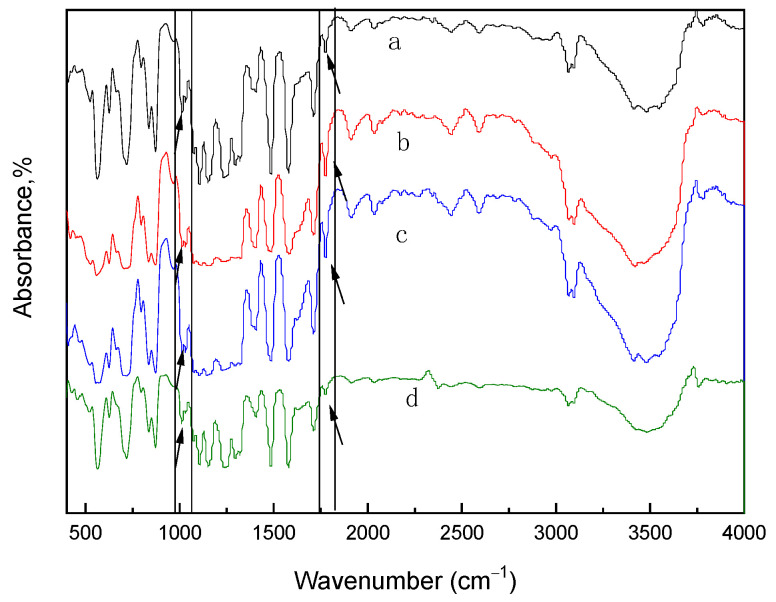
FT-IR spectra for different DS of the SPES/NPHCs blend membranes (at SPES/NPHCs 85:15): (**a**) 15% DS, (**b**) 20% DS, (**c**) 25% DS, and (**d**) 30% DS.

**Figure 3:**
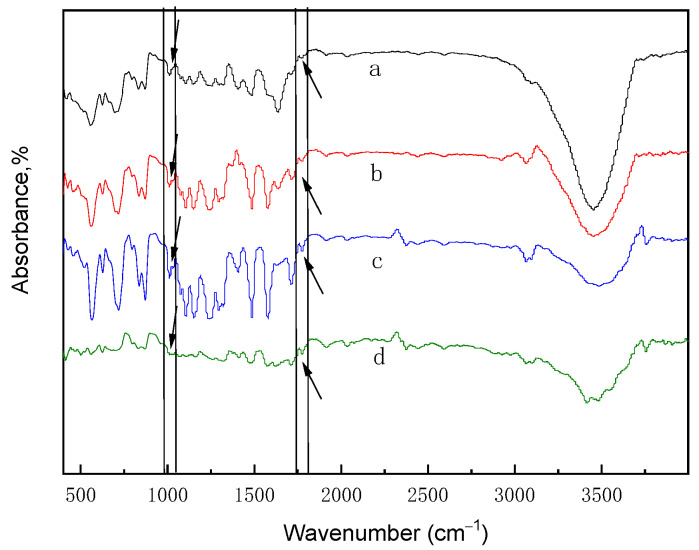
FT-IR spectra for different proportions of the SPES/NPHCs blend membranes (at 30% DS): (**a**) 80: 20, (**b**) 85: 15, (**c**) 90:10, and (**d**) 95:5.

The polymer skeleton is the key component of the membrane. In order to evaluate the polymer structure-morphology, FT-IR spectroscopy was used for the qualitative analysis. Moreover, the chemical nature of the materials, such as the chemical bonds, could be evaluated by identifying the specific absorption peaks for the groups [18,42]. Each compound was analyzed by using FT-IR to determine if the product had been synthesized successfully. Figure 2 and Figure 3 represented the FT-IR of the different SPES/NPHCs-blend membranes. The absorption peak at 1780 cm^−1^ indicated the presence of NPHCs in the polymeric matrix, while absorption peak at 1026 cm^−1^ related to the sulfonic groups confirmed the presence of SPES in the membrane. With the increase of the degree of sulfonation and proportion, the characteristic peak shift amplitude of the structure was not changed significantly, indicating that the force between the two components was stable, which validated that the blend membranes had good compatibility.

**Figure 4:**
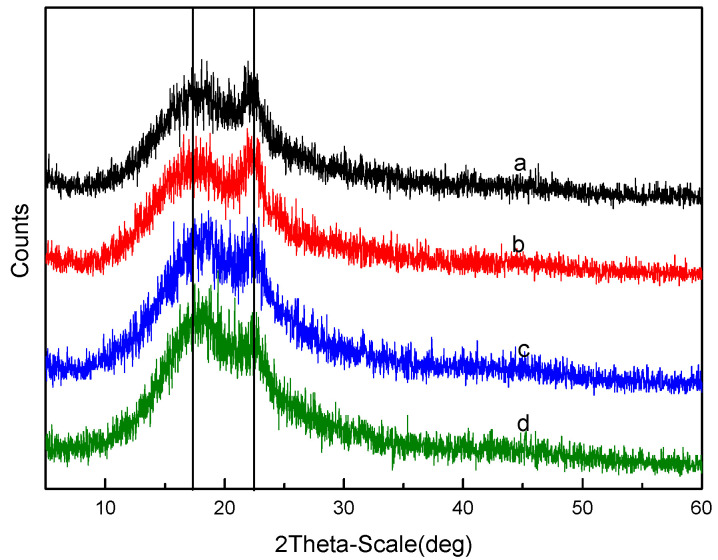
X-ray diffractogram for different DS of the SPES/NPHCs blend membranes (at SPES/NPHCs 85:15): (**a**) 15% DS, (**b**) 20% DS, (**c**) 25% DS, and (**d**) 30% DS.

**Figure 5:**
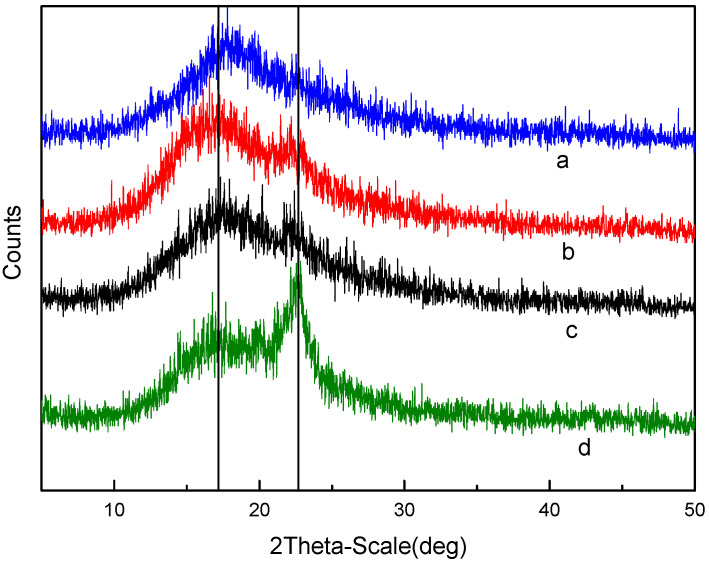
X-ray diffractogram for different proportion of the SPES/NPHCs blend membranes (at 30% DS) (**a**) 80: 20, (**b**) 85:15, (**c**) 90:10, and (**d**) 95:5.

XRD patterns were used to determine the change of the material crystalline nature. The crystalline nature plays a very important role in understanding the substance’s solubility [43,44]. The X-ray diffractograms of the prepared membranes with different DS and SPES/NPHCs ratios are presented in Figure 4 and Figure 5, respectively. The diffraction peaks were offset to different degrees, indicating the interactions between the SPES and NPHCs. With the increase of DS, the peak value decreased (at 2θ = 17°) due to the action of the hydrophilic group -SO_3_H. With the increase of the NPHCs proportion, the peak intensity (at 2θ = 22°) decreased, which indicated the increase of the amorphous properties of the blended film. When the NPHCs proportion exceeded 20%, the modified chitosan destroyed most of the rigid structure.

### 3.3. Optimization of the Procedure by RSM

#### 3.3.1. The Box–Behnken Surface Statistical Design on SPES/NPHCs/MIL-101(Fe) CEMs

The Box–Behnken design (BBD) matrix and the experimental results for the response for the CEMs are presented in Table 1. The matrix was conducted for 17 combinations, consisting of 12 trials and 5 center points for the selected conditions. The response variables and input variables were related by the following second-order polynomial equation:

(5) $$Y_{1}=20.05-9.51\times A+3.46\times B+0.56\times C-0.016\times \mathrm{AB}-0.32\times \mathrm{AC}+0.34\times \mathrm{BC}+2.87\times A^{2}-0.44\times B^{2}+0.041\times C^{2}$$

(6) $$Y_{2}=0.97-0.29\times A+0.16\times B-0.036\times C+0.002375\times \mathrm{AB}+0.012\times \mathrm{AC}+0.003325\times \mathrm{BC}-0.029\times A^{2}-0.03\times B^{2}-0.007758\times C^{2}$$

(7) $$Y_{3}=65.02+3.96\times A-2.83\times B-2.49\times C-0.048\times \mathrm{AB}-0.16\times \mathrm{AC}-0.1\times \mathrm{BC}+0.93\times A^{2}+2.55\times B^{2}+0.41\times C^{2}$$

(8) $${\;Y}_{4}=4.84+0.49\times A-0.003452\times B-0.29\times C-0.042\times \mathrm{AB}-0.005883\times \mathrm{AC}-0.00417\times \mathrm{BC}-0.53\times A^{2}-0.059\times B^{2}-0.025\times C^{2}$$

where A, B and C are the values of the SPES content, DS and MIL-101(Fe) content, respectively.

The significance of the three independent variables for response is usually evaluated by an F-value and *p*-value. Values of “Prob > F” less than 0.0001 indicate the model terms are highly significant. Values of “Prob > F” less than 0.05 indicate the model terms are significant, and vice versa [1]. The predicted R^2^ is an index that indicates how well the model predicts the responses to new observations.

**Figure 6:**
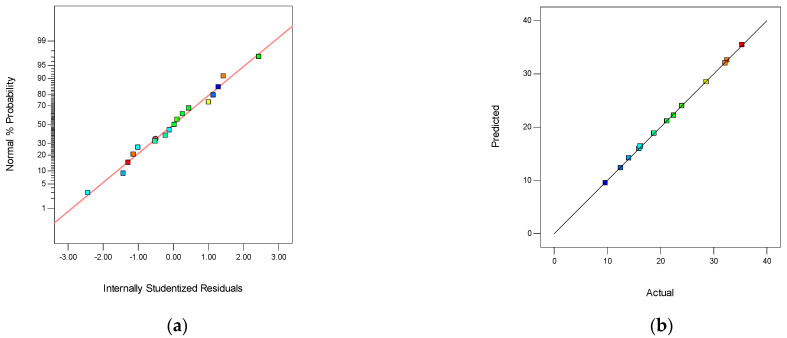
The normality of the residuals and the relationship between the actual and predicted values. (**a**) The normal probability plot of Y_1_, and (**b**) the relationship between the actual and predicted values of *Y*_1_.

The ANOVA results for *Y*_1_ of the quadratic model are presented in Table 2. The model “*p*-value” less than 0.0001 indicated that the matrix response was highly significant. The model “*p*-value” less than 0.05 verified that the matrix response was significant. The value of “Prob > F” less than 0.0001 exhibited that the model terms were highly significant. In this case, the “*p*-value” of A, B, A^2^ was less than 0.001, demonstrating that these three factors were highly significant for Y_1_. The parameters of C, AC, BC, B^2^ were significant for Y_1_. The normal probability plot of studentized residuals is shown in Figure 6a. The data points in this plot were located quite close to the straight line, supporting the significance of the model, and confirming that the assumptions of the analysis were satisfied. The relationship between the actual and predicted values is presented in Figure 6b. Furthermore, a good agreement was observed, indicating that the RSM model was suitable for the data range investigated in this study. The difference between the predicted R_p_^2^ (99.58%) and adjusted R_a_^2^ (99.92%) was 0.0034, demonstrating the high correlation between the observed and the predicted values.

**Figure 7:**
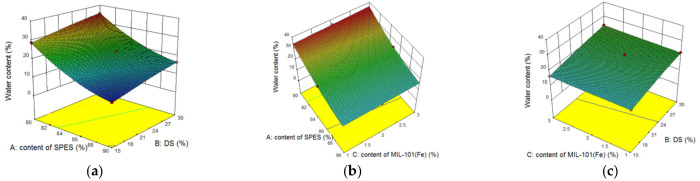
Response surface plot for Y_1_: (**a**) the influence of the DS and content of SPES on the water content of membrane at MIL-101(Fe) content of 2%; (**b**) the influence of the MIL-101(Fe) content and content of SPES on the water content of membrane at DS of 30%; and (**c**) the influence of the MIL-101(Fe) content and DS on the water content of the membrane at a content of SPES of 85%.

The 3D surface plots were graphical diagrams of regression equations showing two factors, while all other factors were maintained at fixed levels. Shown in Figure 7 are the response surface plots showing the influence of the DS, content of SPES and MIL-101(Fe) content for Y_1_. With the increase of MIL-101(Fe) doping amount (from 1% to 3%), the water content of the MMMs increased from 34% to 37%. The addition of the MIL-101(Fe) nanoparticles strengthened the hydrophilicity of the membrane, which was mainly due to the hydrophilic nature of the MIL-101(Fe). In addition, with the addition of hydrophilic MOFs, the hydrophilicity of the membrane was also improved compared with the membrane without MOFs. At the same time, the hydrophilicity of the membrane was also affected by functional groups. There were many hydrophilic functional groups in the membrane that would adsorb water molecules, and the water molecules could have acted as ion transport carriers affecting the separation performance of the membrane. The response surface analysis showed the water content of the membrane increased by about 20% with the increase of DS from 15% to 30%. When the membrane absorbed water and expanded in the water environment, the increase of the water content in the membrane would form ion cluster regions. This was also conducive to reducing the membrane resistance, but it was necessary to avoid an excessive swelling of the membrane due to water absorption, which would reduce the ion selectivity and mechanical strength of the membrane, thus reducing the service life of the membrane. Similarly, the increase of the SPES content meant that the NPHCs content in the membrane decreased, which was more hydrophilic. Comprehensive analysis revealed the WC of the membrane was related to the concentration of the ion-exchange functional groups in the membrane via macromolecular polymers (SPES and NPHCs), and that the impact was greater than from the doping of the MOFs. The increase of the SPES content in the composite membrane also increased the hydrophilic functional groups in the membrane, which in turn affected the separation performance of the membrane.

**Figure 8:**
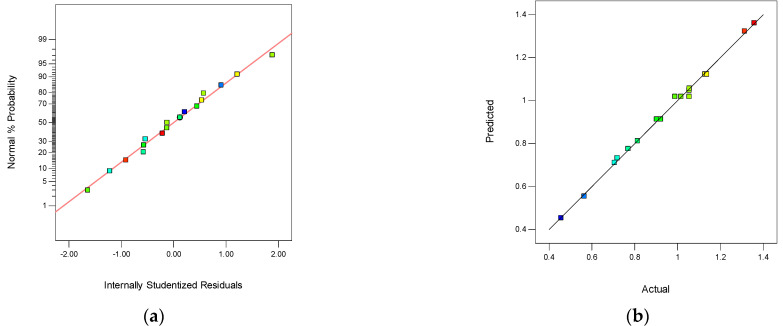
The normality of the residuals and the relationship between the actual and predicted values. (**a**) The normal probability plot of Y_2_, and (**b**) the relationship between the actual and predicted values of Y_2_.

Table 3 shown the ANOVA results for Y_2_ of the quadratic model. The value of “Prob > F” less than 0.0001 indicated that the model terms were highly significant. In this case, the “*p*-value” of A and B was less than 0.001, demonstrating that these two factors were highly significant for Y_2_. The parameters of C, A^2^, and B^2^ were significant for Y_2_. The normal probability plot of studentized residuals is presented in Figure 8a. The data points in this plot were located quite close to the straight line, supporting the significance of the model, and confirming that the assumptions of the analysis were satisfied. The relationship between the actual and predicted values is exhibited in Figure 8b. The RSM model was suitable for evaluating this process. The difference between the predicted R_p_^2^ (98.57%) and adjusted R_a_^2^ (99.25%) was 0.0068, demonstrating the high correlation between the observed and the predicted values. The “Lack of Fit F-value” of 0.23 implied that the Lack of Fit was not significant relative to the pure error.

**Figure 9:**
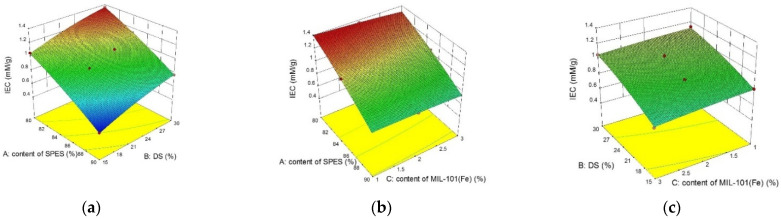
Response surface plot for Y_2_: (**a**) the influence of the DS and content of SPES on IEC of the membrane at a MIL-101(Fe) content of 2%; (**b**) the influence of the MIL-101(Fe) content and content of SPES on IEC of the membrane at a DS of 30%; and (**c**) the influence of the MIL-101(Fe) content and DS on IEC of the membrane at a content of SPES of 85%.

Shown in Figure 9 are the response surface plots given the influence of the DS, content of SPES and MIL-101(Fe) content for Y_2_. The ion exchange capacity (IEC) of the membrane was an important characteristic parameter to quantify the concentration of active functional groups contained in the membrane. Through response surface analysis, when the SPES content and DS of the ion exchange membrane were 85% and 30%, the IEC of the membrane dropped slightly from about 1.15 to 1.1, with an increase of the doping amount of MIL-101(Fe) from 1% to 3%. The possible reason for this was that the addition of the MIL-101(Fe) affected the ion sites (sulfonic acid groups). The data indicated that the water content was directly proportional to the IEC, and both increased with the increase of DS (from 0.76 to 1.15). Therefore, when the water content of the membrane was controlled at an appropriate level, the increase of the IEC effectively reduced the resistance of the membrane. The SPES had a significant impact on the IEC. When the content of the MIL-101 (Fe) and DS were fixed, the IEC decreased from 1.37 to 0.78 with an increase of SPES and a decrease of NPHCs.

**Figure 10:**
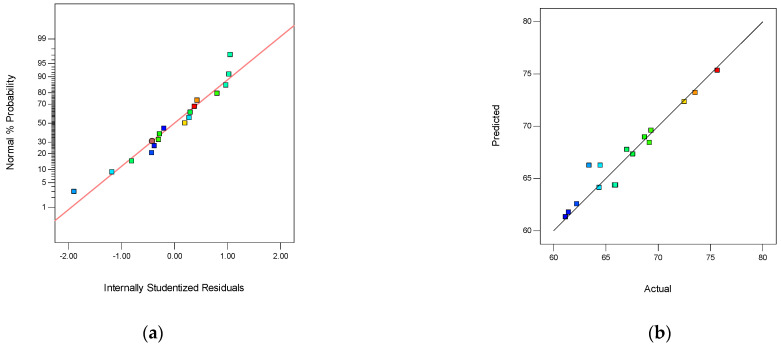
The normality of the residuals and the relationship between the actual and predicted values. (**a**) The normal probability plot of Y_3_, and (**b**) the relationship between the actual and predicted values of Y_3_.

**Figure 11:**
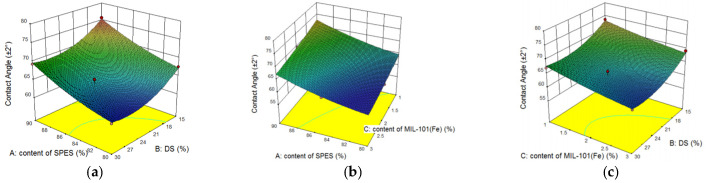
Response surface plot for Y_3_: (**a**) the influence of the DS and content of SPES on the contact angle of the membrane at a MIL-101(Fe) content of 2%; (**b**) the influence of the MIL-101(Fe) content and content of SPES on the contact angle of the membrane at a DS of 30%; and (**c**) the influence of the MIL-101(Fe) content and DS on the contact angle of the membrane at a content of SPES of 85%.

Table 4 shows the ANOVA results for Y_3_ of the quadratic model. The value of “Prob > F” less than 0.05 indicated that the model terms were significant. In this case, the parameters of A, B, C, and B^2^ were significant for Y_3_. The normal probability plot of studentized residuals is shown in Figure 10a. The data points in this plot were located quite close to the straight line, supporting the significance of the model, and confirming that the assumptions of the analysis were satisfied. The relationship between the actual and predicted values is exhibited in Figure 10b. These results verified that the RSM model was a promising strategy for optimizing the preparation of CEMs. The difference between the predicted R_p_^2^ (80.26%) and adjusted R_a_^2^ (83.46%) was 0.032, which illustrated a good agreement between the experimental results and the predicted values. Shown in Figure 11 are the response surface plots showing the influence of the DS, content of SPES and MIL-101(Fe) content for Y_3_. With an increase of MIL-101(Fe) doping (from 1% to 3%), the contact angle reduced by 10%. The contact angle of the membrane decreased by 9% with an increase of DS (from 15% to 30%). This variation could be correlated with the water content analysis.

**Figure 12:**
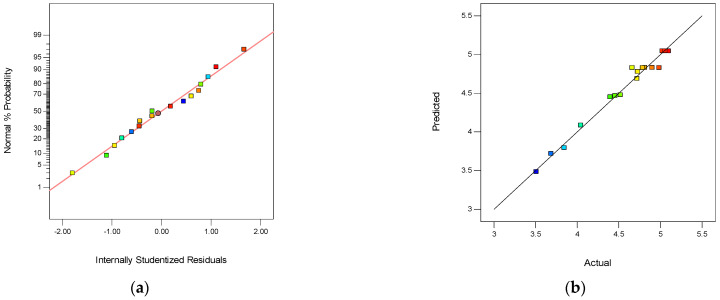
The normality of the residuals and the relationship between the actual and predicted values. (**a**) The normal probability plot of Y_4_, and (**b**) the relationship between the actual and predicted values of Y_4_.

Table 5 presents the ANOVA results for Y_4_ of the quadratic model. The value of “Prob > F” less than 0.0001 indicated that the model terms were highly significant. In this case, the “*p*-value” of A and A^2^ less than 0.001, demonstrated that these two factors were highly significant for Y_4_. The parameter of C was significant for Y_4_. The normal probability plot of studentized residuals is shown in Figure 12a. The data points in this plot were located quite close to the straight line, supporting the significance of the model, and confirming that the assumptions of the analysis were satisfied. The relationship between the actual and predicted values is shown in Figure 12b. It could be observed that the RSM model was a suitable methodology for modeling the synthesizing parameters. The difference between the predicted R_p_^2^ (89.74%) and adjusted R_a_^2^ (95.48%) was 0.0574, demonstrating the high correlation between the observed and the predicted values. The “Lack of Fit F-value” of 0.27 implies the Lack of Fit was not significant relative to the pure error.

**Figure 13:**
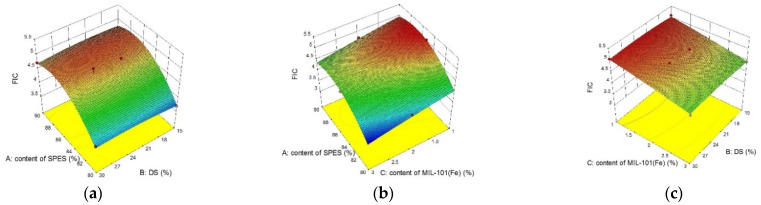
Response surface plot for Y_4_: (**a**) the influence of the DS and content of SPES on FIC of the membrane at a MIL-101(Fe) content of 2%; (**b**) the influence of the MIL-101(Fe) content and content of SPES on FIC of the membrane at a DS of 30%; and (**c**) the influence of the MIL-101(Fe) content and DS on FIC of the membrane at a content of SPES of 85%.

Figure 13 presents the response surface plots showing the influence of the DS, content of SPES and MIL-101(Fe) content for Y_4_. The fixed ion concentration (FIC) of the membrane was an important indicator of the synergistic effect between the membrane water content and the IEC. When the SPES content and DS of the ion exchange membrane were 85% and 30%, respectively, the FIC decreased from 5 to 4.5. This variation could be correlated with the IEC analysis. From the 3D response surface curve of Y_4_, the FIC decreased as the content of the NPHCs in the MMMs. When the NPHCs content was 15%, the FIC was 4.55, which was caused by the content of the NPHCs.

#### 3.3.2. Effect of MIL-101(Fe) Content on MIL-101(Fe) Hybrid Membrane

With the result in the response surface plot for Y_1_, Y_2_, Y_3_ and Y_4_, the effects of MIL-101(Fe) concentrations of 0 wt%, 1 wt%, 2 wt% and 3 wt% at casting solution temperatures of 60 °C were studied. Table 6 displays the results of the performance test for these membranes. As mentioned above, the organic linkers provided a good affinity with the MOFs and organic polymers, encouraging the formation of non-covalent bonds between them. Noncovalent interactions enhanced the interface compatibility, promoting the improvement on the properties of the membrane’s active layer without any adverse effect on its selectivity. Moreover, the casting solution became relatively viscous with a MIL-101(Fe) concentration from 0.0 wt% to 3.0 wt%, the water content increased from 21.9% to 29.21%, and the contact angle decreased from 67.96° to 60.45° [45,46,47,48]; however, the excessively viscous casting solution had a detrimental influence on the formation of thin membranes, and it also promoted the formation of membranes with uneven thicknesses, causing maldistribution of the membrane pores. As the MIL-101(Fe) concentration increased to 3.0 wt%, part of the MIL-101(Fe) was not well dispersed in the casting solution and led to a blockage of the membrane pore. Therefore, 2.0 wt% of MIL-101(Fe) was determined to be the optimum concentration.

### 3.4. Membrane Structure—Thermal STABILITY

**Figure 14:**
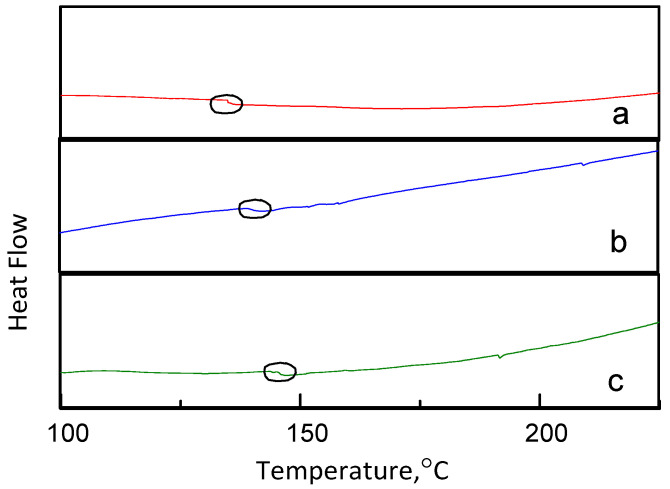
DSC curves for different ratios of SPES/NPHCs/MIL-101(Fe) blend membranes (at SPES/NPHCs 85:15): (**a**) 0 wt%, (**b**) 1 wt%, and (**c**) 2 wt%.

DSC is a commonly used thermal analysis tool that helps to find the glass transition temperature (Tg) and analyzes the thermal stability of the material. Tg of the polymer is an important criterion for the compatibility of polymer components. The completely miscible polymer blend had a single Tg, while the immiscible polymer blend had a plurality of Tg. As shown in Figure 14, each DSC trace showed a single Tg, indicating good miscibility between the SPES, NPHCs and MIL-101(Fe) due to the formation of van der Waals interactions. In Figure 14, with the increase of MIL-101(Fe) content, the Tg of the blend membrane was decreased, which meant that the thermal stability of the membrane decreased with the increase of MIL-101(Fe) content. The difference in Tg values was observed due to the polymer domain interactions produced by the different forms of various MIL-101(Fe) contents and the differences in the mechanical properties of the blend membranes.

### 3.5. Membrane Structure-Morphology

**Figure 15:**
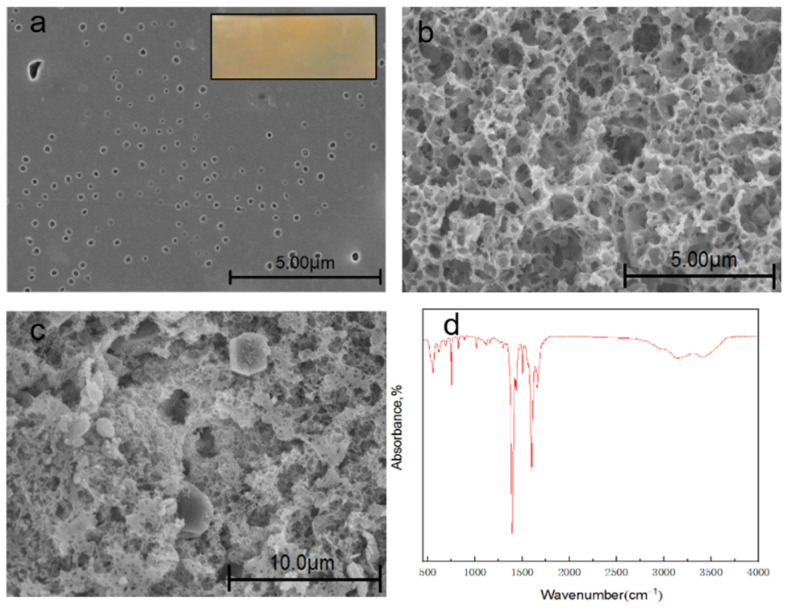
Characterizations of MMMs (at 85 wt% SPES and 30% DS): (**a**) SEM image of membrane containing 13 wt% NPHCs and 2 wt% MIL-101(Fe) (surface); (**b**) SEM image of membrane containing 15 wt% NPHCs (cross−sectional); (**c**) SEM image of membrane containing 13 wt% NPHCs and 2 wt% MIL-101(Fe) (cross−sectional); and (**d**) FT-IR spectra for membrane containing 2 wt% MIL-101(Fe) and 13 wt% NPHCs (surface).

The microscopic morphology of the membranes helps to determine the importance of membrane in the mechanisms of permeability and selectivity. Thus, the morphology of the prepared membranes was evaluated by scanning electron microscopy. Figure 15 presents SEM images of the blend membranes with surface, section, and partial section, respectively. The surface morphology of the membrane showed it was uniform and smooth without visible flaws, which indicated that the blend membranes had good compatibility. The distribution of tunnels was still relatively uniform. Under the premise of ensuring the mechanical strength and thermal stability of the membrane, the porous ion exchange membrane had a significant effect on the diffusion behavior and exhibited excellent performance in the electrodialysis process. Furthermore, the distribution of MIL-101(Fe) was relatively uniform. Pore size of the membrane is an important parameter for desalination process. Therefore, the pore size distribution of the membrane was measured by Nano Measurer for size distribution analysis to evaluate the permeability and selectivity of the membrane. Figure S8 presents the average pore size distribution for membranes with different contents (SPES/NPHCs/MIL-101(Fe) at 30% DS. With the increase of NPHCs content, the pore size had a significant increase. When the large amount of NPHCs was added (at 20%), the average pore size distribution was two to three times larger than the 15 wt% NPHCs. This result was attributed to the NPHCs tending to form clusters. It was noteworthy that the pore size did not have a significant increase due to the organic linkers present in the MIL-101(Fe) structures which provided a better affinity with the MIL-101(Fe) and organic polymers. The data of burst strength test was presented in Figure S9. A significant reduction in burst strength of the membrane after an increase in the NPHCs was observed, and the SPES/N-phthaloyl chitosan/MIL-101(Fe) membrane showed almost the same burst strength value as the SPES/N-phthaloyl chitosan membrane. The mechanical properties illustrated that the NPHCs could lead to a changing of porosity which reduces mechanical strength. Nevertheless, the moderate loading of MIL-101(Fe) had little influence on the mechanical strength of the membrane.

### 3.6. Water Content, Ion-Exchange Capacity and Hydrophilicity

A large number of hydrophilic functional groups of the ion exchange membrane adsorb water molecules and water molecules act as ion transport carriers, which directly affect the membrane separation performance. Hence, it was important to select membranes with the appropriate water content. The water content increased with sulfonation degree (Table 6) due to a hydrophilic sulfonic acid group. As a chitosan derivative, the NPHCs contained polar functional groups like hydroxyl, and ether groups that could improve the water content. With the introduction of MIL-101(Fe), the water attracting capacity of the membrane increased. These results indicated that the water content was proportional to the IEC. Likewise, the NPHCs, sulfonation degree and MIL-101(Fe) all affected the membrane ion exchange performance. The sulfonic acid groups and NPHCs provided active sites for the proper interaction between ions and the membrane surface, thereby enhancing the feasibility of the ion exchange. The contact angle was used to characterize the outermost changes in membrane modification to evaluate the hydrophilicity of the membrane. The contact angle data of different membranes are presented in Table 6. The contact angle decreased with an increase of MOF content, indicating an increase in hydrophilicity of the blend membrane. This could be ascribed to the agglomeration of the MOF. The excessive MOF content in the membrane (>3 wt%) caused an uneven dispersion of the powder. Accordingly, a MOF content of 2 wt% in the membrane did not affect the hydrophilicity of the membranes.

### 3.7. Membrane Porosity and Desalination

The porous ion exchange membranes had a good property. The diffusion effect depended on the presence of active ion exchange groups and pores throughout the membrane. Therefore, to investigate the change in porosity of the prepared membranes under different reaction conditions, the diffusion coefficient was measured.

**Figure 16:**
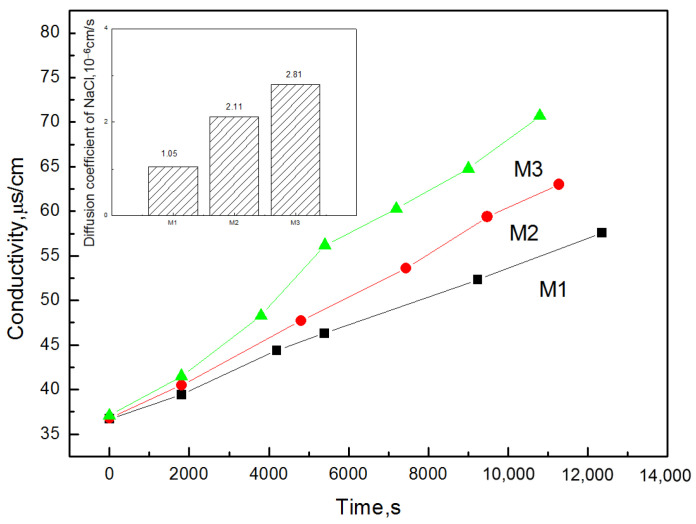
The effect of MOF content on NaCl diffusion behavior of the prepared ion exchange membranes (at SPES/NPHCs 85:15): (**M1**) 1 wt%; (**M2**) 2 wt%; and (**M3**) 3 wt%.

**Figure 17:**
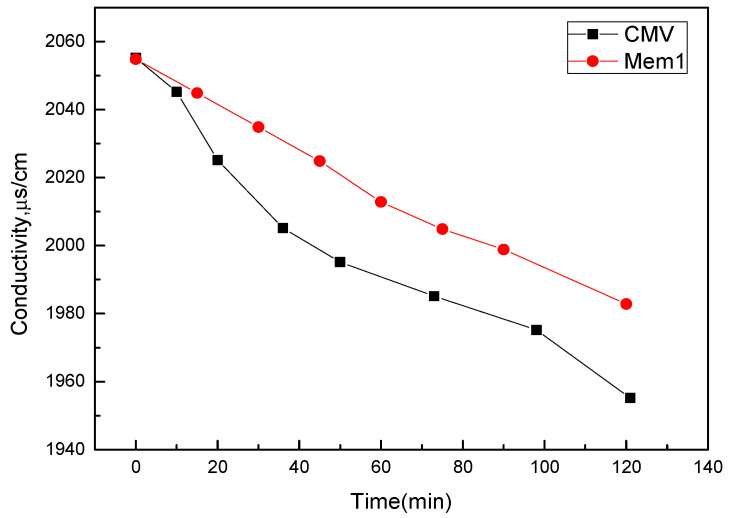
The effect of MOF additive on NaCl desalination behavior of the prepared ion exchange membranes (at SPES/NPHCs 85:15): CMV, 2 wt%; mem 1, 0 wt%.

**Figure 18:**
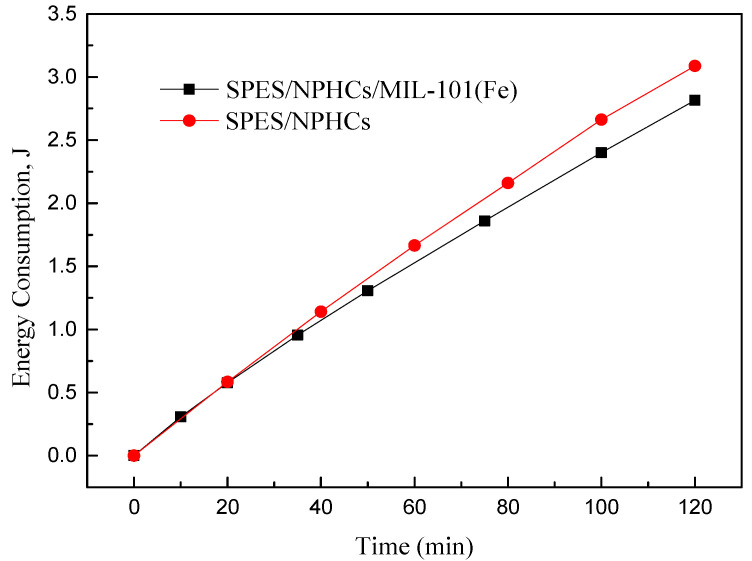
Energy consumption in electrodialysis desalination experiment with SPES/NPHCs/MIL-101(Fe) and SPES/NPHCs.

As seen in Figure 16, the NaCl diffusion behavior increased with the increase of MOF content, which meant that the porosity of the composite membranes was improved by the effect of the MOF. In Figure 17, the desalination rate of the CEMs increased with the MOF content increasing. The increase in MOF content caused the membrane porosity to accumulate more electrolyte ions, thus, the diffusion of Na^+^ was strengthened, which was consistent with the above-mentioned IEC experiments; however, the mechanical properties of the membrane reduced as the MOF content increased, which resulted in the diffusion coefficient of the membrane being 4 wt% which could not be measured. The membrane M_2_ was facilitated, and when comparing it with other membranes, this study gained more satisfactory results. With the effect of the MOF additive on the NaCl diffusion behavior of the prepared CEMs, the MOF could, therefore, improve the desalination performance of the membrane compared with the membrane without a MOF. The energy consumption is presented in Figure 18. It can be observed that with a 2% MIL-101(Fe) loading into the membrane, the desalination rate of the membranes improved to 136% and the energy consumption reduced to 90%.

## 4. Conclusions

In this study, we reported the synthesis of hybrid cation exchange membranes (CEMs) by embedding MIL-101(Fe) into a SPES/NPHCs polymetric backbone by the RSM model. The properties of the CEMs observed by SEM, FTIR, XRD and DSC verified the successful reaction and good compatibility between SPES, NPHCs and MIL-101(Fe). As a result, the MIL-101(Fe) was a good pore-filler for the SPES matrix due to a good interaction with the polymer matrix and improvement in the hydrophilic nature of the membrane, which enhanced the membrane’s conductivity and stability. According to a regression model and ANOVA, the optimal synthetic factors, such as DS, the ratio of SPES and NPHCs and the content of MIL-101(Fe), were determined to be 30%, 85:15 and 2%, respectively, and the corresponding desalination rate of the CEMs improved to 136%, while the energy consumption reduced to 90%. Therefore, based on the above data, the construction of SPES/NPHCs/MIL101-(Fe) hybrid membranes using a RSM statistical model is a promising approach in the design of high-performance cation exchange membrane for desalination applications.

## Figures and Tables

**Table 1:** BBD matrix for three factors and the observed results for the response variable.

Assay	A-SPES Content (%)	B-DS (%)	C-MIL-101(Fe) Content (%)	Response			
				Y_1_: WC (%)	Y_2_: IEC (mM/g)	Y_3_: Contact Angle (±2°)	Y_4_: FIC
1	85	25	2	21.23	1.02	65.92	4.79
2	80	30	2	35.33	1.36	61.44	3.85
3	80	25	1	32.48	1.31	64.37	4.04
4	80	20	3	32.16	1.13	61.15	3.51
5	85	20	2	18.80	0.92	64.48	4.90
6	85	25	2	21.16	1.05	65.84	4.98
7	90	30	2	16.30	0.77	69.33	4.72
8	90	25	1	14.05	0.71	72.51	5.02
9	85	15	1	15.95	0.81	73.55	5.10
10	90	15	2	9.65	0.46	75.67	4.73
11	85	30	3	24.01	1.06	62.23	4.40
12	85	15	3	16.14	0.72	69.17	4.45
13	85	30	1	22.48	1.14	67.01	5.06
14	85	20	2	18.75	0.90	63.40	4.81
15	90	20	3	12.49	0.56	68.71	4.52
16	80	15	2	28.61	1.05	67.58	3.69
17	85	25	2	21.20	0.99	65.96	4.66

**Table 2:** ANOVA results for Y_1_ of the RSM of the reduced quadratic model.

Source	Sum of Squares	df	Mean Square	F Value	*p*-Value Probe > F	
Model	860.62	9	95.62	2338.01	<0.0001	highly significant
A-content of SPES	723.83	1	723.83	17,697.52	<0.0001	
B-DS	104.97	1	104.97	2566.48	<0.0001	
C-content of MIL-101(Fe)	2.42	1	2.42	59.05	0.0001	
AB	0.001	1	0.001	0.025	0.8795	
AC	0.36	1	0.36	8.92	0.0203	
BC	0.45	1	0.45	11.07	0.0126	
A^2^	33.59	1	33.59	821.17	<0.0001	
B^2^	0.63	1	0.63	15.38	0.0057	
C^2^	0.007	1	7.019 × 10^−^^3^	0.17	0.6911	
Residual	0.29	7	0.041			
Lack of Fit	0.28	4	0.071	50.67	0.0044	
Pure Error	0.004	3	0.001			
Cor Total	860.91	16				

**Table 3:** ANOVA results for Y_2_ of the RSM of the reduced quadratic model.

Source	Sum of Squares	df	Mean Square	F Value	*p*-Value Probe > F	
Model	0.96	9	0.11	237.40	<0.0001	highly significant
A-content of SPES	0.70	1	0.70	1549.59	<0.0001	
B-DS	0.22	1	0.22	490.62	<0.0001	
C-content of MIL-101(Fe)	0.010	1	0.010	22.90	0.0020	
AB	0.00002	1	2.256 × 10^−5^	0.050	0.8290	
AC	0.00005	1	4.879 × 10^−4^	1.09	0.3319	
BC	0.00004	1	4.422 × 10^−5^	0.098	0.7628	
A^2^	0.00351	1	3.512 × 10^−3^	7.82	0.0266	
B^2^	0.00300	1	3.001 × 10^−3^	6.68	0.0362	
C^2^	0.000245	1	2.461 × 10^−4^	0.55	0.4832	
Residual	0.00314	7	4.490 × 10^−4^			
Lack of Fit	0.00074	4	1.841 × 10^−4^	0.23	0.9055	not significant
Pure Error	0.00241	3	8.022 × 10^−4^			
Cor Total	0.96	16				

**Table 4:** ANOVA results for Y_3_ of the RSM of the reduced quadratic model.

Source	Sum of Squares	df	Mean Square	F Value	*p*-Value Probe > F	
Model	259.33	9	28.81	9.97	0.0031	significant
A-content of SPES	125.49	1	125.49	43.43	0.0003	
B-DS	69.96	1	69.96	24.21	0.0017	
C-content of MIL-101(Fe)	48.50	1	48.50	16.78	0.0046	
AB	0.009	1	0.009	0.003	0.9570	
AC	0.094	1	0.094	0.032	0.8623	
BC	0.041	1	0.041	0.014	0.9081	
A^2^	3.54	1	3.54	1.22	0.3052	
B^2^	21.02	1	21.02	7.27	0.0308	
C^2^	0.70	1	0.70	0.24	0.6370	
Residual	20.23	7	2.89			
Lack of Fit	19.64	4	4.91	25.22	0.0121	
Pure Error	0.58	3	0.19			
Cor Total	279.56	16				

**Table 5:** ANOVA results for Y_4_ of the RSM of the reduced quadratic model.

Source	Sum of Squares	df	Mean Square	F Value	*p*-Value Probe > F	
Model	3.83	9	0.43	38.59	<0.0001	significant
A-content of SPES	1.91	1	1.91	172.93	<0.0001	
B-DS	1.044 × 10^−4^	1	1.044 × 10^−4^	9.463 × 10^−^^3^	0.9252	
C-content of MIL-101(Fe)	0.67	1	0.67	60.86	0.0001	
AB	6.943 × 10^−^^3^	1	6.943 × 10^−^^3^	0.63	0.4536	
AC	1.246 × 10^−4^	1	1.246 × 10^−4^	0.011	0.9183	
BC	6.956 × 10^−5^	1	6.956 × 10^−5^	6.308 × 10^−^^3^	0.9389	
A2	1.16	1	1.16	104.95	<0.0001	
B2	0.011	1	0.011	1.03	0.3440	
C2	2.635 × 10^−^^3^	1	2.635 × 10^−^^3^	0.24	0.6399	
Residual	0.077	7	0.011			
Lack of Fit	0.020	4	5.049 × 10^−^^3^	0.27	0.8835	not significant
Pure Error	0.057	3	0.019			
Cor Total	3.91	16				

**Table 6:** The physical properties of membranes with different ratios of (SPES+NPHCs)/MOFs (at 30% DS).

Membrane	Water Content %	IEC (mM·g^−1^)	Contact Angle(°)	FIC
(SPES + NPHCs): MOFs 100:0	21.90	1.14	67.96 ± 2	5.21
(SPES + NPHCs): MOFs 99:1	22.41	1.29	65.43 ± 2	5.76
(SPES + NPHCs): MOFs 98:2	24.62	1.37	63.21 ± 2	5.56
(SPES + NPHCs): MOFs 97:3	26.73	1.54	60.45 ± 2	5.76

## Data Availability

Not applicable.

## Supplementary Materials

The following supporting information can be downloaded at: https://www.mdpi.com/article/10.3390/membranes12020144/s1, Figure S1: Schematic representation of sulfonation polyethersulfone; Figure S2: Schematic representation of the prepared N-phthloyl chitosan; Figure S3: The setup used for the measurement of diffusion coefficient; Figure S4: The setup used for the measurement of desalination; Figure S5: XRD patterns of MIL-101 (Fe) nanoparticles stability test: (a) deionized water; (b) Hydrochloric acid; (c) Sodium hydroxide solution; Figure S6: The SEM image of MIL-101(Fe): standard form; Figure S7: FTIR spectroscopy of MIL-101(Fe) and standard MIL-101(Fe); Figure S8: Pore size distribution for membranes with different content (SPES /NPHCs/MIL-101(Fe) at 30% DS; Figure S9: Burst strength for membranes with different content (SPES /NPHCs/MIL-101(Fe) at 30% DS.
