# Supplementary material for: Fabrication of a Cation-Exchange Membrane via the Blending of SPES/N-Phthaloyl Chitosan/MIL-101(Fe) Using Response Surface Methodology for Desalination
Source: Membranes (Basel). 2022 Jan 25;12(2):144. doi: 10.3390/membranes12020144 (PMC8880603; doi:10.3390/membranes12020144)
Supplement: Supplementary file 1 [file membranes-12-00144-s001.zip › membranes-1560895-supplementary.pdf]

membranes

Supplementary Materials

Fabrication of a Cation-Exchange Membrane via the Blending of SPES/N-Phthaloyl Chitosan/MIL-101(Fe) Using Response Surface Methodology for Desalination

Xiaomeng Wang [1,†], Qun Wang [1,†], Mengjuan Zhao [1], Lu Zhang [1], Xiaosheng Ji [2,*], Hui Sun [3], Yongchao Sun [4,5], Zhun Ma [1,*], Jianliang Xue [6] and Xueli Gao [4]

1 College of Chemical and Biological Engineering, Shandong University of Science and Technology, Qingdao 266590, China; 15764237673@163.com (X.W.); 18561555725@163.com (Q.W.); zmj971830@163.com (M.Z.); luluzh5709@163.com (L.Z.)
2 Sanya Institute of Oceanology, Chinese Academy of Sciences, Sanya 572000, China
3 State Key Laboratory of High-efficiency Utilization of Coal and Green Chemical Engineering, Ningxia University, Yinchuan 750021, China; sunhui@nxu.edu.cn
4 Key Laboratory of Marine Chemistry Theory and Technology, Ministry of Education; College of Chemistry and Chemical Engineering, Ocean University of China, Qingdao 266100, China; yongchao_sun@163.com (Y.S.); gxl_ouc@126.com (X.G.)
5 School of Chemical Engineering, Dalian University of Technology, Dalian 116024, China
6 College of Safety and Environmental Engineering, Shandong University of Science and Technology, Qingdao 266590, China; ll-1382@163.com
* Correspondence: jixiaoshen@zju.edu.cn (X.J.); skdmaz919@sdust.edu.cn (Z.M.)
† These authors contributed equally to this work.

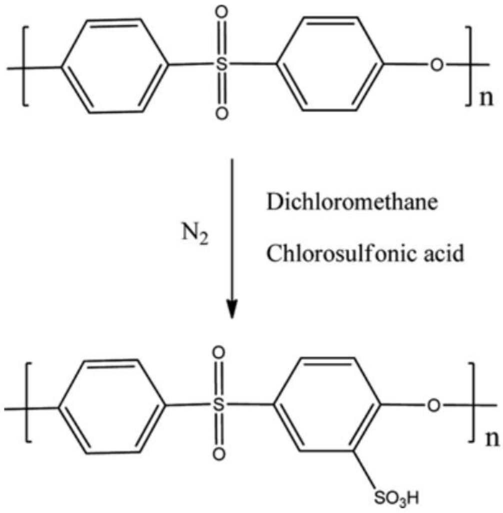

Figure S1. Schematic representation of sulfonation polyethersulfone.

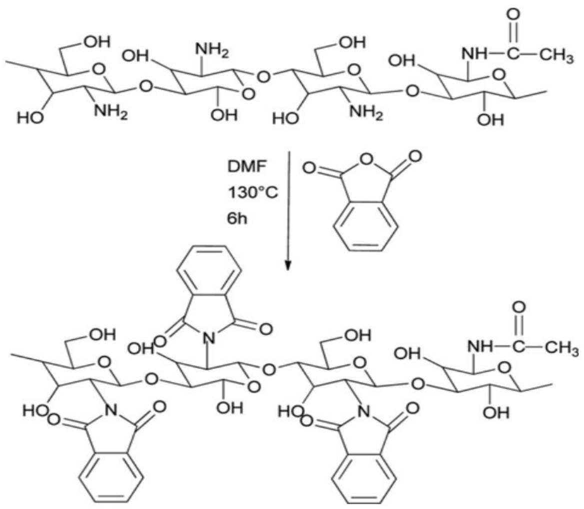

Figure S2. Schematic representation of the prepared N-phthloyl chitosan.

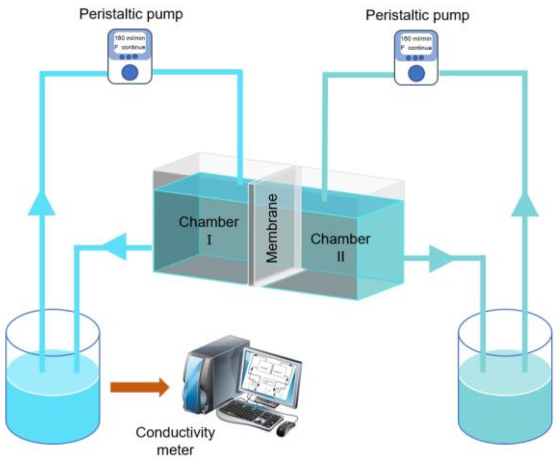

Figure S3. The setup used for the measurement of diffusion coefficient.

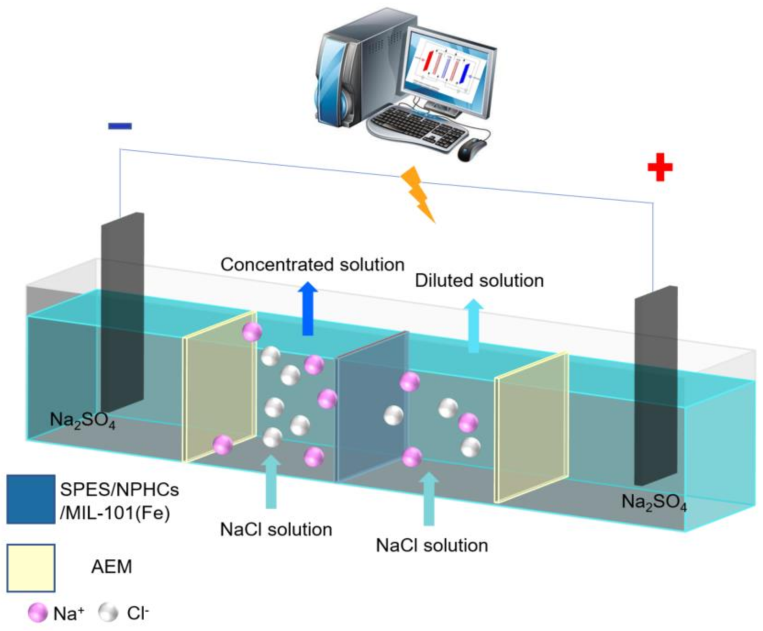

Figure S4. The setup used for the measurement of desalination.

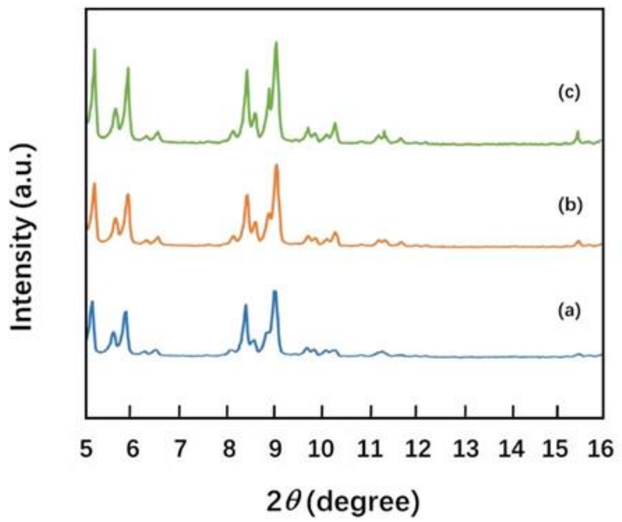

Figure S5. XRD patterns of MIL-101 (Fe) nanoparticles stability test :(a) deionized water; (b) Hydrochloric acid; (c) Sodium hydroxide solution.

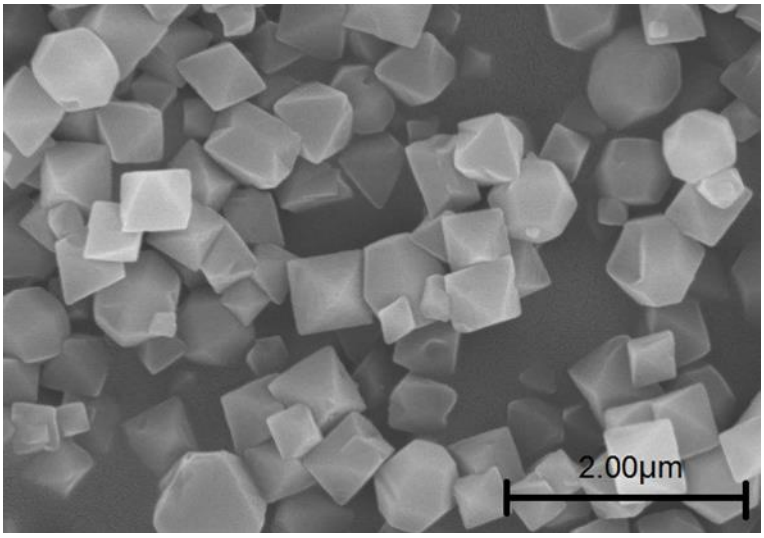

Figure S6. The SEM image of MIL-101(Fe): standard form.

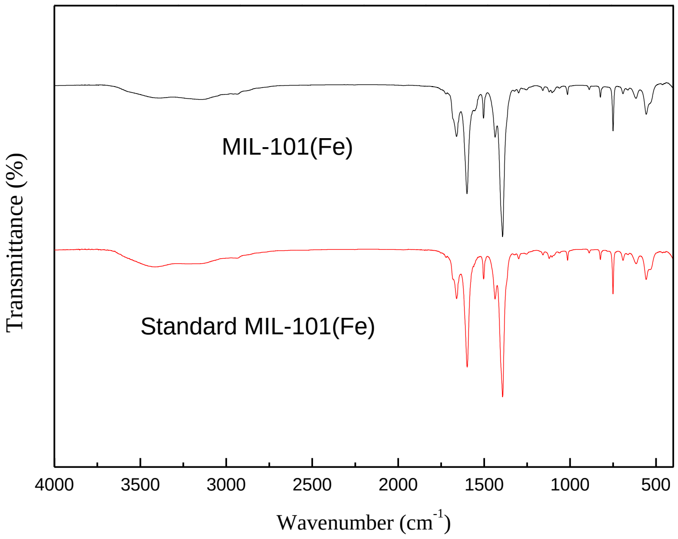

Figure S7. FTIR spectroscopy of MIL-101(Fe) and standard MIL-101(Fe).

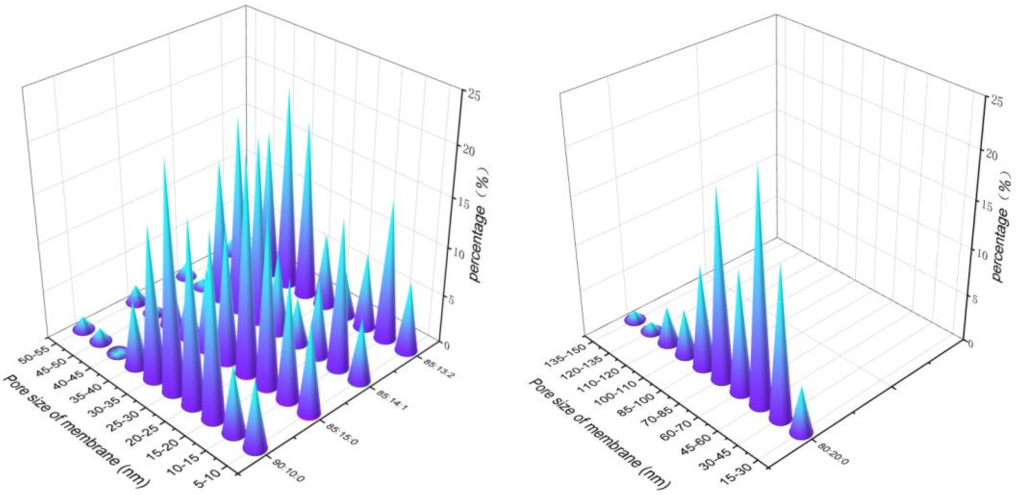

Figure S8. Pore size distribution for membranes with different content (SPES /NPHCs/MIL-101(Fe) at 30% DS.

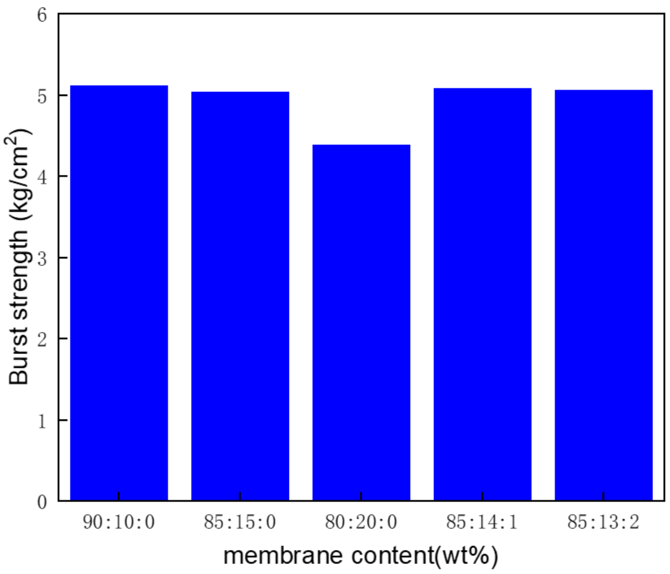

Figure S9. Burst strength for membranes with different content (SPES /NPHCs/MIL-101(Fe) at 30% DS.
